# Risk factors of poor prognosis and impairment of activities of daily living in patients with hemorrhagic gastroduodenal ulcers

**DOI:** 10.1186/s12876-020-01580-w

**Published:** 2021-01-06

**Authors:** Junya Arai, Jun Kato, Nobuo Toda, Ken Kurokawa, Chikako Shibata, Shigeyuki Kurosaki, Kazuyoshi Funato, Mayuko Kondo, Kaoru Takagi, Kentaro Kojima, Takamasa Ohki, Michiharu Seki, Kazumi Tagawa

**Affiliations:** Department of Gastroenterology, Mitsui Memorial Hospital, 1 Kanda-Izumi-cho, Chiyoda-ku, Tokyo, 101-8643 Japan

**Keywords:** Gastroduodenal ulcer, Elderly, Prognosis, Activity of daily living

## Abstract

**Background:**

Impairment of activities of daily living (ADL) due to hemorrhagic gastroduodenal ulcers (HGU) has rarely been evaluated. We analyzed the risk factors of poor prognosis, including mortality and impairment of ADL, in patients with HGU.

**Methods:**

In total, 582 patients diagnosed with HGU were retrospectively analyzed. Admission to a care facility or the need for home adaptations during hospitalization were defined as ADL decline. The clinical factors were evaluated: endoscopic features, need for interventional endoscopic procedures, comorbidities, symptoms, and medications. The risk factors of outcomes were examined with multivariate analysis.

**Results:**

Advanced age (> 75 years) was a significant predictor of poor prognosis, including impairment of ADL. Additional significant risk factors were renal disease (odds ratio [OR] 3.43; 95% confidence interval [CI] 1.44–8.14) for overall mortality, proton pump inhibitor (PPIs) usage prior to hemorrhage (OR 5.80; 95% CI 2.08–16.2), and heart disease (OR 3.05; 95% CI 1.11–8.43) for the impairment of ADL. Analysis of elderly (> 75 years) subjects alone also revealed that use of PPIs prior to hemorrhage was a significant predictor for the impairment of ADL (OR 8.24; 95% CI 2.36–28.7).

**Conclusion:**

In addition to advanced age, the presence of comorbidities was a risk of poor outcomes in patients with HGU. PPI use prior to hemorrhage was a significant risk factor for the impairment of ADL, both in overall HGU patients and in elderly patients alone. These findings suggest that the current strategy for PPI use needs reconsideration.

## Background

Hemorrhagic gastroduodenal ulcers (HGUs) are the most common cause of upper gastrointestinal bleeding. Although most HGUs can be safely treated with medications and/or endoscopic procedures [1], some patients with HGU have a poor prognosis owing to comorbidities [2] and medications for anticoagulation [3]. Indeed, mortality from HGU is still high, with reported rates up to 10% [4].

Several reports have evaluated the risk factors of mortality of HGU [5–9]. A previous systematic review has shown that older age, comorbidity, shock, and delayed treatment are independent risk factors of mortality [5]. Another study revealed that endoscopic features with a high-risk Forrest classification (Forrest I, IIa, and IIb) are prognostic indicators of mortality [6].

In addition to mortality, impairment of activities of daily living (ADL) is also a very important prognostic issue, particularly in elderly subjects, leading to longer hospitalization and higher treatment cost [10]. Moreover, lowering of ADL is closely correlated with mortality in elderly subjects [11]. Changes in ADL associated with disease outcome have been evaluated mainly in diseases that directly influence patients’ physical functions, such as hip fracture and stroke [12, 13]. In this regard, there have been no studies that have evaluated which risk factors of HGU are associated with impairment of ADL. This is important in both clinical care of patients who are identified to be at risk of ADL impairment after HGU and planning of clinical and social care services. This is especially relevant in care of the elderly since previous reports have revealed that advanced age is a risk factor of poor outcomes for HGU patients [5, 7, 8].

In this study, the risk factors of poor prognosis, including mortality and impairment of ADL, were examined in patients with HGU. In particular, the prognosis of elderly HGU patients (≥ 75 years old) was evaluated in comparison to that of non-elderly (< 75 years old) patients, since previous reports have revealed that advanced age is a risk factor of poor outcomes for HGU patients [5, 7, 8].

## Methods

### Patients

A retrospective cohort study was performed using the database of endoscopy and medical record reviews from Mitsui Memorial Hospital, a secondary emergency hospital in Tokyo, Japan, between August 2004 and December 2017.

We extracted the data of patients who had evidence of gastroduodenal ulcers identified by endoscopy from the endoscopic database. Those who showed hemorrhagic implications with symptoms including black stool, hematemesis, and/or anemia in medical records were included. If patients experienced two or more episodes of HGU during the study period, the first episode alone was included in the analysis. Patients who were confirmed as malignant ulcers by pathological diagnosis were excluded. Those with lower ADL on admission due to comorbidities including malignancy and dementia were not excluded because we focused on each patient’s change of ADL, but patients who had resided in care facilities before hospitalization were excluded when examining the impairment of ADL. In this study, both gastric and duodenal ulcers were included in HGUs because management of these diseases is similar including gastric acid inhibition, endoscopic hemostasis, and eradication of *Helicobacter pylori* [14].

Our policy for management of HGU was as follows. Endoscopic hemostasis was generally performed for HGU with Forrest I or IIa according to the Forrest classification [15]. If the index endoscopic hemostasis was unsuccessful with bleeding continuing, surgery or angioembolization was considered. The second look endoscopy was done before start of eating for patients with high-risk HGU such as large size and Forrest I or II. EGD for biopsy to rule out gastric cancer was also performed.

The following clinical factors were evaluated as they have been shown to be associated with the severity of bleeding or prognosis of HGU [5, 6]. Endoscopic features and need for interventional endoscopic procedures were collected from endoscopic database. The information of comorbidities, symptoms, and medications were reviewed with medical records. The evaluated endoscopic features included locations of HGU (fundus, gastric body, gastric angle, antral zone, anterior wall of duodenal bulb, posterior wall of duodenal bulb, and second portion of duodenum), type of hemorrhage using the Forrest classification [15], size of ulcer (≥ 20 mm or not), and the number of ulcers (≥ 2 ulcers or not). Interventional endoscopic procedures indicate endoscopic hemostasis including clipping, argon plasma coagulation, heat probe, sprinkling of thrombin, injection of hypertonic saline-epinephrine solution, and injection of epinephrine. The following comorbidities were recorded: heart disease, pulmonary disease, stroke, diabetes mellitus, liver disease, renal disease, and history of gastroduodenal ulcers before the study period. The evaluated symptoms included black stool, syncope, and hematemesis. Medication use prior to hemorrhage was documented for anticoagulants, antiplatelets, nonsteroidal anti-inflammatory drugs (NSAIDs), cyclooxygenase-2 inhibitors, corticosteroids, proton pump inhibitors (PPIs), and H2 receptor antagonists (H2RAs).

This analysis defined patients ≥ 75 years of age as elderly, and the clinical backgrounds and outcomes were compared between patients < 75 years old and those ≥ 75 years old. The primary outcomes were overall mortality, mortality within 30 days after occurrence of hemorrhage, and impairment of ADL. Admission to a care facility or the need for home adaptations such as a care bed, during the hospitalization period were defined as ADL decline. When estimating the impairment of ADL, 13 patients who resided in care facilities before hospitalization were excluded. The secondary outcomes were rebleeding, hospitalization periods, and need for transfusion.

The Ethics Committee of Mitsui Memorial Hospital approved this retrospective study, which conformed to the provisions of the Declaration of Helsinki (as revised in Fortaleza, Brazil, October 2013). For this type of study formal consent is not required.

### Statistical analysis

Continuous data are presented as mean ± standard deviation, and categorical data as actual frequencies and percentages. Continuous data were compared with the Student’s t-test. Comparisons of categorical data between the groups were performed with the chi-square test or Fisher’s exact test as appropriate. Independent risk factors of primary outcomes were identified using multivariate logistic regression analysis based on parameters with *p* < 0.1 in univariate analysis.

A *p *value < 0.05 was considered statistically significant, and odds ratios (ORs) with 95% confidence intervals (CIs) were determined. All statistical analyses were performed with EZR (Saitama Medical Center, Jichi Medical University, Saitama, Japan), which is a graphical user interface for R (The R Foundation for Statistical Computing, Vienna, Austria). More precisely, EZR is a modified version of R commander designed to add statistical functions frequently used in biostatistics.

## Results

### Patient characteristics

In total, 582 patients (425 men and 157 women; mean age, 66.6 ± 14.4 years) were diagnosed with HGU at least once during the study period, and the first episode of these patients were analyzed. Table 1 shows the evaluated clinical factors of all patients. The following variables were significantly higher in patients ≥ 75 years old than patients < 75 years old: HGU in the gastric body [47% vs. 33%, *p* = 0.001], HGU > 20 mm (33% vs. 23%, *p* = 0.02), comorbidities (heart disease [34% vs. 21%, *p* < 0.001], pulmonary disease [12% vs. 7%, *p* = 0.044], and stroke [14% vs. 5%, *p* < 0.001]), symptoms of hematemesis (36% vs. 24%, *p* = 0.0038), and antiplatelet use (38% vs. 19%, *p* < 0.001). Meanwhile, HGU in the anterior wall of duodenal bulb [14% vs. 25%, *p* = 0.0024], HGU with the Forrest classification IIa (27% vs. 37%, *p* = 0.02), and history of gastroduodenal ulcers [16% vs. 27%, *p* = 0.0023] were less likely observed in patients ≥ 75 years old.

**Table 1:** Baseline characteristics of HGU patients

Characteristics	Non-elderly (n = 390)	Elderly (n = 192)	*p* value
Patient number	390	192	
Sex (male/female)	309/81	116/76	< 0.001
Age (mean ± SD)	59.2 ± 11.3	81.8 ± 5.0	< 0.001
Weekend hospitalization	90 (23%)	38 (20%)	0.43
Location of HGU			
Fundus	13 (3%)	5 (2%)	0.8
Gastric body	127 (33%)	90 (47%)	0.001
Gastric angle	72 (19%)	30 (16%)	0.47
Antral zone	51 (13%)	30 (16%)	0.48
Anterior wall of duodenal bulb	97 (25%)	26 (14%)	0.0024
Posterior wall of duodenal bulb	30 (8%)	12 (6%)	0.64
Second part of duodenum	6 (2%)	5 (3%)	0.52
Endoscopic features			
Forrest classifications			
Ia	32 (8%)	17 (9%)	0.92
Ib	65 (17%)	39 (20%)	0.34
IIa	145 (37%)	52 (27%)	0.02
IIb	38 (10%)	19 (10%)	1
IIc	27 (7%)	23 (12%)	0.059
III	83 (21%)	42 (22%)	0.96
Size > 20 mm	91 (23%)	63 (33%)	0.02
> 2 ulcers	167 (43%)	96 (50%)	0.12
Need for interventional endoscopic procedure	233 (60%)	98 (51%)	0.057
Comorbidities			
Heart disease	83 (21%)	66 (34%)	< 0.001
Pulmonary disease	26 (7%)	23 (12%)	0.044
Stroke	18 (5%)	26 (14%)	< 0.001
Diabetes	82 (21%)	34 (18%)	0.41
Liver disease	21 (5%)	19 (10%)	0.062
Renal disease	46 (12%)	18 (9%)	0.46
History of GU	107 (27%)	30 (16%)	0.0023
Symptoms			
Hematemesis	94 (24%)	69 (36%)	0.0038
Black stool	315 (81%)	144 (75%)	0.14
Syncope	15 (4%)	10 (5%)	0.59
Medications			
Anticoagulation	29 (7%)	23 (12%)	0.099
Antiplatelet	74 (19%)	73 (38%)	< 0.001
NSAIDS	61 (16%)	43 (22%)	0.059
COX-2 inhibitor	3 (1%)	4 (2%)	0.23
Corticosteroids	14 (4%)	7 (4%)	1
PPI	42 (11%)	24 (13%)	0.63
H2RA	24 (6%)	14 (7%)	0.73

*ADL* activity of daily living, *COX* cyclooxygenase, *GU* gastroduodenal ulcers, *H2RA* histamine 2 receptor antagonist, *NSAIDs* nonsteroidal anti-inflammatory drugs, *PPI* proton pump inhibitor, *SD* standard deviation

### Primary and secondary outcomes of patients < 75 years old versus those ≥ 75 years old

Table 2 shows the outcomes of the two groups. Overall mortality (8% vs. 4%, *p* = 0.048), impairment of ADL (7% vs. 1%, *p* < 0.001) and need for transfusion (67% vs. 51%, *p* < 0.001) were frequently observed in patients ≥ 75 years old than in patients < 75 years old. In addition, patients ≥ 75 years old had significantly longer hospitalization period (23.1 ± 28.2 days vs. 16.0 ± 29.3 days, *p* = 0.0056).

**Table 2:** Outcomes of patients

Outcomes	Non-elderly (n = 390)	Elderly (n = 192)	*p* value
Overall mortality	15 (4%)	15 (8%)	0.048
Mortality within 30 days after hemorrhage	7 (2%)	9 (5%)	0.058
Impairment of ADL	5 (1%)	13 (7%)	< 0.001
Hospitalization period (days)	16.0 ± 29.3	23.1 ± 28.2	0.0056
Rebleeding	62 (16%)	28 (15%)	0.77
Transfusion	200 (51%)	128 (67%)	< 0.001

When estimating the impairment of ADL, 13 patients who had resided in care facilities before hospitalization were excluded

*ADL* activity of daily living

### Independent risk factors of primary outcomes in multivariate logistic regression analysis

**Fig. 1:**
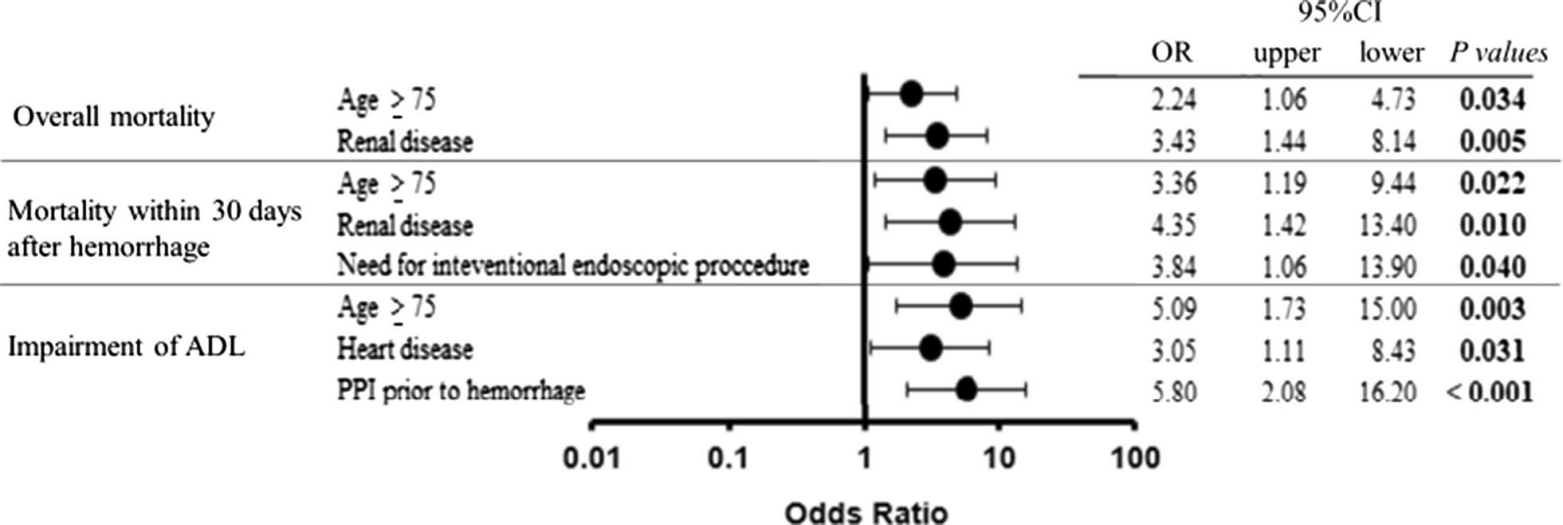
Independent risk factors of primary outcomes in multivariate logistic regression analysis. *ADL* activity of daily living, *PPI* proton pump inhibitor, *OR* odds ratio, *CI* confidence interval. When estimating the impairment of ADL, 13 patients who had resided in care facilities before hospitalization were excluded

Multivariate analysis revealed that age (≥ 75 years) (OR 2.24; 95% CI 1.06 –4.73), and renal disease (OR 3.43; 95% CI 1.44–8.14) were independent and significant risk factors for overall mortality. The risk factors of mortality within 30 days after hemorrhage were age (≥ 75 years) (OR 3.36; 95% CI 1.19–9.44), need for interventional endoscopic procedures (OR 3.84; 95% CI 1.06–13.9), and renal disease (OR 4.35; 95% CI 1.42–13.4). Finally, the risk factors of impairment of ADL were use of PPI prior to hemorrhage (OR 5.80; 95% CI 2.08–16.2), heart disease (OR 3.05; 95% CI 1.11–8.43), and age (≥ 75 years) (OR 5.09; 95% CI 1.73–15.0) (Fig. 1).

### Independent risk factors of primary outcomes for elderly patients in multivariate logistic regression analysis

**Fig. 2:**
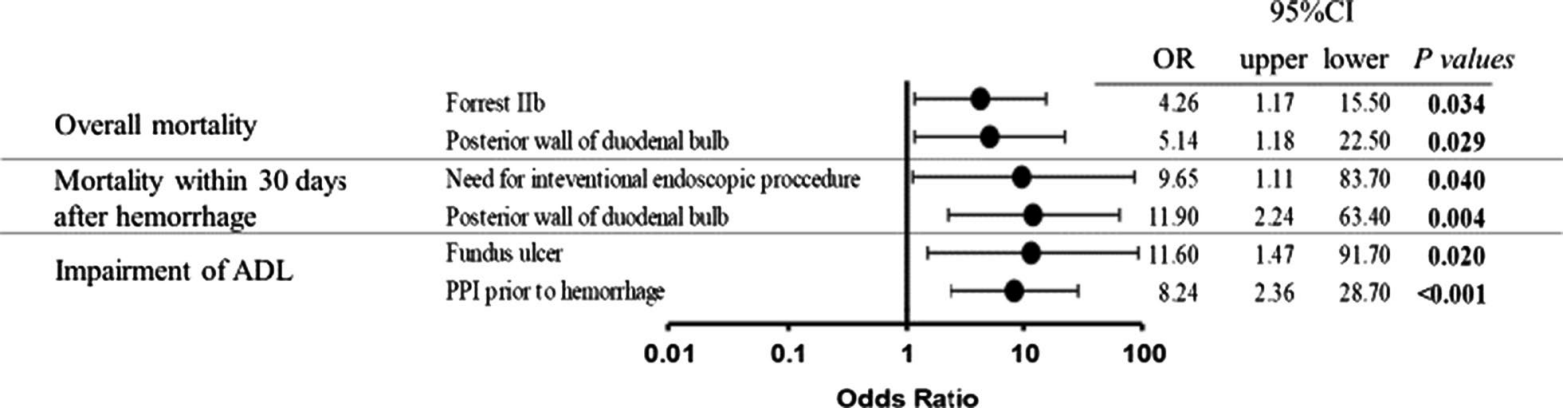
Independent risk factors of primary outcomes for elderly patients in multivariate logistic regression analysis. *OR* odds ratio, *CI* confidence interval. When estimating the impairment of ADL, 6 patients who had resided in care facilities before hospitalization were excluded

The prognostic factors in elderly subjects were analyzed. The independent and significant risk factors for overall mortality were Forrest IIb (OR 4.26; 95% CI 1.17–15.5), and ulcer location of posterior wall of duodenal bulb (OR 5.14; 95% CI 1.18–22.5). The risk factors of mortality within 30 days after hemorrhage were need for interventional endoscopic procedure (OR 9.65; 95% CI 1.11–83.7), and location of posterior wall of duodenal bulb (OR 11.9; 95% CI 2.24–63.4). The risk factors of impairment of ADL were fundus ulcer (OR 11.6; 95% CI 1.47–91.7), and use of PPI prior to hemorrhage (OR 8.24; 95% CI 2.36–28.7) (Fig. 2).

## Discussion

In this study, the risk factors of poor prognosis and impairment of ADL in patients with HGU were examined. Advanced age, comorbidities (renal disease and heart disease), need for interventional endoscopic procedures, and use of PPI prior to hemorrhage were identified as independent risk factors for worse outcomes. Moreover, we particularly focused on the impairment of ADL by HGU and found that PPI use prior to hemorrhage was a significant risk factor that impaired ADL both in overall subjects and in elderly subjects alone.

Advanced age was a risk factor of overall mortality and mortality within 30 days after hemorrhage; this result is in line with several previous studies [5, 7, 8]. Advanced age was also an independent risk factor of impairment of ADL. Iwatsuka et al. showed that the elderly patients (> 70 years old) with gastrointestinal bleeding were less likely to return home after hospital discharge than non-elderly patients (< 70 years old) (5% vs. 21%, *p* < 0.001) [7]. However, their study included many types of gastrointestinal bleedings, from benign ulcers to malignant tumors. In contrast, we analyzed the risk factors of impairment of ADL in patients with HGU alone, and elderly subjects with gastrointestinal bleeding were at risk even with benign disease. The attribute of limited physiologic reserves, sarcopenia and frailty in elderly is probably responsible. In this regard, more attention should be paid to rehabilitation and prehabilitation for those subjects [16].

The need for interventional endoscopic procedures was a risk factor of mortality within 30 days after hemorrhage. Endoscopic procedures are generally performed for HGU with risky appearance, including Forrest I and II. In this regard, and consistent with our results, Lanie et al. showed that endoscopic features of Forrest I and II are prognostic indicators of mortality [6]. In contrast, Giese et al. showed that those Forrest scores are not significantly associated with death within 30 days, and rebleeding [17]. They discussed the discrepancy from Lanie’s results based on the timing of endoscopy, because they always performed endoscopy within 6 h of admission. In our hospital, when severe bleeding is implied by symptoms, vital signs, laboratory data, medications, or comorbidities, EGD is performed as soon as possible after admission, but otherwise, EGD is performed electively. Earlier endoscopic interventions, regardless of the severity of HGU, might improve the prognosis of patients with HGU. In this regard, recent guidelines suggested stratifying patients based on the Blatchford score and adopting a risk-stratified management and recommended urgent inpatient endoscopy (≤ 12 h of admission) in the high-risk group [18].

In this study, HGU patients with renal disease had worse prognosis, and similar results have also been reported previously [8, 19]. Renal disease in general is poor prognosticator for many diseases and pathologies. In particular, bleeding leads to acute on chronic kidney injury and hence drives poor outcomes [20]. This result may also be partly due to lower hemoglobin levels in patients with chronic kidney disease because anemia is reportedly the risk factor of mortality for patients with a higher risk of stroke or cardiovascular events [21, 22]. In addition, uremic platelet function impairment is a potential cause of the higher risk for ulcer bleeding complications [23]. Uremic platelet dysfunction involves von Willebrand factor with platelet membrane glycoproteins Ib and IIb to IIIa, which is not normalized after dialysis [24]. Furthermore, anticoagulation use during hemodialysis may contribute to the risk [19].

The presence of heart disease was one of the risk factors of impairment of ADL. It has been reported that when patients have some cardiac abnormality, such as valvular heart disease or underlying left ventricular dysfunction, severe anemia can cause hyper-output heart failure [25] resulting in long hospitalization and impairment of ADL. Eikelboom et al. revealed that there is a strong, consistent, temporal, and dose-related association between bleeding and poor prognosis in patients who have a history of acute coronary syndromes [26]. They discussed that major bleeding is likely to lead physicians to discontinue effective antithrombotic drugs such as aspirin, clopidogrel, heparin, low-molecular-weight heparin, and warfarin, which in turn could increase the risk of ischemic heart disease, stroke, and cardiovascular death. Furthermore, it is commonly known that heart disease is one of the risk factors of sarcopenia that could cause impairment of ADL. Consequently, sarcopenia in patients with HGU might affect the results [27, 28].

PPI use was identified as a risk factor of impairment of ADL. Most of the patients were prescribed PPI for the prevention of peptic ulcers caused by NSAIDs (21%) and antiplatelets (including aspirin) (47%) or due to a previous history of peptic ulcers (9%). A previous report showed that approximately 5% of patients with a history of ulcer bleeding who continued to take both NSAIDs and PPI still had recurrent ulcers after 6 months [29]. Another study revealed that 19%–26% of patients with similar situations had endoscopically assessed gastroduodenal ulcers at 6 months, and the risk factors of ulcer recurrence included age ≥ 75 and comorbidity (heart diseases, stroke, cirrhosis, respiratory diseases, renal diseases, and diabetes) [30]. Thus, ulcer development as a result of NSAIDs under PPI treatment is not rare, and based on our results, such ulcers may result in poor clinical outcomes. Although the precise reasons are unknown, a possible risk of osteoporosis and dementia owing to taking PPIs may also be associated with the poor outcomes of PPI users [31, 32]. Moreover, the relatively low dose of PPIs in Japanese patients (e.g. esomeprazole 10–20 mg/day) could not sufficiently prevent the development of ulcers as a result of NSAIDs.

In the elderly, the locations of HGU (fundus and the posterior wall of the duodenum) were identified as independent risk factors of worse outcomes, but the presence of comorbidities was not. Localization on the posterior wall of the duodenal bulb has been shown to be a risk factor for adverse outcome in bleeding peptic ulcers [33]. Furthermore, the morbidity rate of fundus ulcers in elderly people is known to be higher than that in non-elderly people [34]. The seriousness of ulcers or bleeding is likely to be associated with mortality for elderly patients. Indeed, Chow et al. reported that the risk of death was increased in elderly patients (age > 80) with features of severe bleeding (transfusion > 4 units, ulcer size > 2 cm, hematemesis, and melaena) at presentation [8]. In this regard, the finding that Forrest IIb was the risk of overall mortality was of interest. Although patients with Forrest I or Forrest IIa ulcer always received endoscopic hemostasis, those with Forrest IIb did not. The difference in treatment strategy between the stages of ulcers might contribute to the result [35]. The fact that comorbidities were not identified as a prognostic factor in the elderly is not surprising because most elderly patients have at least one comorbidity.

The present study has several strengths. First, our study included a relatively high number of patients with a number of variables. Second, we evaluated each comorbidity separately instead of using the Charlson Comorbidities Index, which resulted in the identification of independent risk factors.

This study has several limitations that should be recognized. First, this study was a retrospective and single center study and there could be some biases. In particular, relatively homogenous socio-cultural status and limited health infrastructure in our hospital might affect the results. Second, the patients in our study might have had more severe heart and renal diseases than patients in other studies because our hospital is a tertiary center of heart diseases and renal diseases in the eastern region of Tokyo. Third, we defined ADL impairment as admission to care facilities after hospital discharge or requirement of home modification for rehabilitation at home. Although a previous report applied similar definitions in the estimation of ADL [7], such definitions are not validated. In order to evaluate the changes of ADL more precisely, validated measurement scores such as the Pune-FAAT scale or the MDS-derived ADL scale should be used. Fourth, the patients’ information about ADL after discharge may be insufficient due to limited access. Finally, the characteristics of our patients did not include the *Helicobacter pylori* infection profile; however, as far as we know, no previous study has shown that *Helicobacter pylori* is a risk factor of poor prognosis of HGU.

## Conclusion

Advanced age was a potent predictor of poor outcomes in patents with HGU, and additional risk factors of poor outcomes were different between overall HGU patients and elderly patients alone. The presence of comorbidities was a risk factor in the overall patients, and in particular heart disease was a risk of impairment of ADL. In elderly patients, the location of ulcers was associated with outcomes. Furthermore, PPI use prior to hemorrhage was a significant predictor of impairment of ADL, both in overall patients and in elderly patients alone. Although PPI use has generally been recommended for the prevention of ulcer development, such a policy may have room for reconsideration.

## Data Availability

The datasets used and/or analyzed during the current study are available from the corresponding author on reasonable request.

## Abbreviations

ADL: Activity of daily living
HGU: Hemorrhagic gastroduodenal ulcers
EGD: Esophagogastroduodenoscopy
PPI: Proton pump inhibitor
NSAIDs: Nonsteroidal anti-inflammatory drugs
OR: Odds ratio
CI: Confidence interval
